# Maternity healthcare providers’ self-perceptions of well-being during COVID-19: A survey in Tshwane Health District, South Africa

**DOI:** 10.4102/phcfm.v14i1.3034

**Published:** 2022-01-12

**Authors:** Sarie Oosthuizen, Anne-Marie Bergh, Antonella Silver, Refilwe Malatji, Vivian Mfolo, Tanita Botha

**Affiliations:** 1 Department of Family Medicine, Faculty of Health Sciences, University of Pretoria, Pretoria, South Africa; 2 Research Centre for Maternal, Fetal, Newborns and Child Health Care Strategies, Faculty of Health Sciences, University of Pretoria, Pretoria, South Africa; 3 South African Medical Research Council, Research Unit for Maternal and Infant Health Care Strategies, Faculty of Health Sciences, University of Pretoria, Pretoria, South Africa; 4 District Clinical Specialist Team, Tshwane District Health Services, Tshwane, South Africa; 5 Department of Statistics, Faculty of Natural and Agricultural Sciences, University of Pretoria, Pretoria, South Africa

**Keywords:** COVID-19, maternity healthcare workers, mental health, support, fear, anxiety, stress, depression, anger

## Abstract

**Background:**

Mental health manifestations such as depression and anxiety disorders became more marked during the coronavirus disease 2019 (COVID-19) pandemic as frontline healthcare workers struggled to maintain high-quality intrapartum care and essential health services.

**Aim:**

This study aimed to identify maternity healthcare providers’ self-perceptions of changes in their feelings of mental well-being.

**Setting:**

Ten midwife obstetric units and the labour wards of four district hospitals in Tshwane Health District, South Africa.

**Methods:**

We conducted an anonymous, cross-sectional survey amongst a convenience sample of 114 maternity healthcare workers to gauge the changes in healthcare workers’ experience and perceptions of well-being during the COVID-19 pandemic. Four items measured the perceived changes on a scale of 0–10 for the periods before and during COVID-19, respectively, namely feelings of fear or anxiety, stress, depression and anger.

**Results:**

The majority of participants were professional nurses (37%) and advanced midwives (47%). They reported a significant change in well-being from before the pandemic to during the pandemic with regard to all four items (*p* < 0.0001). The biggest ‘before-during’ difference was in perceptions of fear or anxiety and the smallest difference was in perceptions of anger. A framework was constructed from the open-ended responses to explain healthcare workers’ understanding and perceptions of increased negative feelings regarding their mental well-being.

**Conclusion:**

The observed trends in the changes in healthcare workers’ self-perceptions of their mental well-being highlight the need for further planning to build resilient frontline healthcare workers and provide them with ongoing mental health support and improved communication pathways.

## Introduction

Coronavirus disease 2019 (COVID-19) presented to the world at the start of 2020, was declared a pandemic by the World Health Organization (WHO) and rapidly evolved into a global health emergency. Frontline healthcare workers (HCWs) were confronted with the challenging task of balancing the accelerated pace of the pandemic, the reality of medical countermeasures and the continued provision of essential services.^1,2,3^ The shifting health-system environment and an inability to re-organise themselves put frontline HCWs at greater risk for their own physical and mental health.^4,5,6^

Although HCWs were expected to be resilient in their response to emergency care, the pandemic saw an increase in mental health manifestations such as depression, anxiety disorders, post-traumatic stress disorder (PTSD) and peritraumatic dissociation.^3,7,8,9,10^ These conditions were more marked in female HCWs and nurses,^9,11,12,13^ – groups which were identified as being at greater risk for mental health conditions as a result of family and community factors. Poor communication with supervisors, colleagues diagnosed with COVID-19 and working with infected patients (sometimes without sufficient personal protective resources) led to greater isolation,^12^ overall higher anxiety levels,^14^ stress, distress and burnout.^12,14,15,16^ Fear of and actual transmission of the infection to family members and perceived stigma from relatives and society^12,16^ were additional triggers that led HCWs to feel unsupported, unsafe and traumatised by their environments.^5,17^

Whilst ethics and equity principles highlight the need to protect and maintain all essential health services in low- and middle-income countries (LMICs), these countries have embarked on priority setting based on their own health contexts.^3,18,19^ The additional tasks associated with screening and testing for COVID-19, community awareness activities and contact tracing have exacerbated shortages of health resources and the workload of already overburdened providers.^20,21^ These challenges have also affected essential maternity services negatively.^22^ There have been calls for changes to health-systems functioning to maintain high quality intrapartum care, whilst simultaneously trying to minimise the risk of system collapse.^21^ At the same time, the need has been expressed to protect frontline maternity care workers and build resilience – the ability to resist, absorb, bounce back or recover and learn from the effects of COVID-19^23,24^ – in order to be able to provide quality care.^25,26^

South Africa and Tshwane Health District experienced the same trade-offs between managing the pandemic, reducing financial fallout and supporting struggling HCWs, whilst maintaining basic healthcare services for pregnant women, children and people living with HIV/AIDS.^19^ During support visits to health facilities in Tshwane as part of the CLEVER Maternity Care programme – a programme to improve respectful quality obstetric care^27^ – midwives expressed fears for their own safety, anxiety about their comorbidities and anger about the increased workload and a shortage of personal protective equipment (PPE). Internal arguments as to who should care for confirmed COVID-19 patients or patients under investigation (PUIs) led to additional tension and disrupted harmonious relations in some labour wards.

Because of concerns about maternity healthcare providers’ physical and mental health, a survey was conducted to identify self-perceptions of changes in their feelings of mental well-being as a result of the pandemic. The information obtained will enable the district management to better coordinate mental health support for frontline workers.

## Methods

### Study design and setting

We conducted a cross-sectional, anonymous survey in 14 CLEVER facilities in Tshwane Health District from 18 September 2020 to 03 December 2020. These facilities included 10 midwife obstetric units (MOUs) and 4 district hospitals (DHs).

### Study population and sampling strategy

A convenience sample of healthcare providers working in maternity units in the CLEVER facilities were recruited on the days the CLEVER team members visited a facility. The number of participants per DH ranged between 7 and 16 and between 4 and 8 per MOU.

### Data collection

A quick paper-based, self-reporting tool in English that would not take HCWs away from their service duties was developed to rapidly inform the development of appropriate support strategies in the district. Thirteen HCWs familiar with the context commented on format, length, content and the feasibility of generating useful findings for administering the tool. Maternity HCWs received a copy of the questionnaire from CLEVER team members during monthly or biweekly support visits and the anonymously completed questionnaires were placed in a special collection envelope.

In addition to demographic items (age, gender, designation and years of employment at a CLEVER facility), the questionnaire contained two items related to HCWs’ feelings of well-being before and during the COVID-19 pandemic, and two items on service delivery. This article reports on HCWs’ self-reports on their mental well-being; the experience of service provision will be reported separately.

The first ‘well-being’ item comprised a set of four widely used terms from the literature (fear or anxiety, stress, depression, anger) with an analogue scale of 0–10 for rating perceptions of own emotional state and feelings of well-being before COVID-19 compared with how participants felt during the pandemic (How have your feelings changed since the start of the COVID-19 pandemic?). The instructional example scale included the following explanation of the scale: 0 indicated no negative feeling (‘None’), 4 was labelled as a ‘Mild’ negative feeling, 8 was labelled as ‘Severe’ and 10 as ‘Unbearable’. The second item was an open-ended question in which participants could describe and explain the reasons for the change in their feelings. A further question on the support needs of HCWs was included in the analysis with regard to mental health support needs.

### Data analysis

After capturing and cleaning the data on Microsoft Excel, HCWs who had been employed at a particular health facility for less than one year were excluded (*n* = 21). The reason for exclusion being that these workers had not been at the health facility long enough to be able to draw a comparison between their feelings before and after the advent of COVID-19 pandemic. The excluded group also included student and community service nurses with short attachments.

Data were analysed using the R statistical software package version 3.6.3.^28^ Frequencies, proportions, means (standard deviation [s.d.]) and medians (Q1; Q3) were calculated. No analysis was carried out on the relationship with gender because there was only one male respondent. For the four items on participants’ perceptions of well-being before and during the COVID-19 pandemic, median ‘before’ scores and ‘during’ scores were calculated out of 10 for each item, as well as an overall ‘before’ and ‘during’ well-being score combining responses for the four items. The numerical difference between the ‘before’ and ‘during’ scores was also calculated.

The Shapiro–Wilk test indicated that the quantitative data were not to be normally distributed. The Wilcoxon signed rank test was used to determine whether a significant difference existed between the perceptions of well-being when comparing the results before and during the COVID-19 pandemic. When comparing results between the different demographic categories, the Mann–Whitney U test and the Kruskal–Wallis H test were used, followed by post hoc analysis with a Bonferroni adjustment where further pairwise analysis was needed. The relationship between continuous variables (numerical age and years of employment) and perception changes was established using Spearman’s rank correlation. All tests were performed at a 5% level of significance.

Responses to the open-ended question were analysed inductively for content. Researchers familiarised themselves with the content of the responses and a code book was developed through a consensus-seeking process. All team members contributed to the interpretation of the data.

### Ethical considerations

This study was conducted under the umbrella of a larger maternity care project called CLEVER Maternity Care. The Research Ethics Committee of the Faculty of Health Sciences approved the research protocol as an amendment to the CLEVER protocol (787/2018). The study had the support of the Tshwane District Management Team, which gave permission for HCWs to take part. Study participants completed the questionnaire voluntarily and anonymously. Mental health support for facilities and individual HCWs in need was available through the employee wellness team and the occupational health and safety nurse practitioner in the district.

## Results

### Participant characteristics

Reponses of a total of 114 questionnaires were analysed. There comprised 65 MOU respondents, ranging between 4 and 11 per facility and 49 DH respondents, ranging between 7 and 16 per hospital. The majority of the participants were advanced midwives (*n* = 54; 47.4%) and registered professional nurses (*n* = 43; 37.7%). The remainder were managers (*n* = 8; 7.0%), medical officers (*n* = 3; 2.6%) and enrolled nurses (*n* = 2; 1.8%) or nursing assistants (*n* = 3; 2.6%). One participant did not specify a designation. Seven of the eight managers worked in DHs. Fewer respondents in DHs were registered nurses (*n* = 14; 28.6%) than in MOUs (*n* = 29; 44.6%).

Participants’ age ranged between 25 years and 68 years, with a mean age of 42.63 (±10.71) years. The participants were divided into four groups on the basis of age: 25–30 years, 31–40 years, 41–50 years and > 50 years of age. Ages were fairly equally distributed across three of the four groups (26% – 30%), with the age group 25–30 years having about half the number of respondents (14.2%) compared with the other groups because of its shorter age span. Although the mean ages of participants in MOUs and DHs were similar (43.58 vs 43.68), the MOUs had a larger percentage of participants in the age groups 31–40 years (28.1%) than DHs (20.4%) and a smaller number in the 41–50 years age group (24.6% vs 30.6%). Overall, there was no significant difference between the ages of participants from MOUs and DHs (*p* = 0.9398).

We divided the length of participants’ employment at the study facility into four groups for easier overview. One quarter of respondents fell into in the employment group 1 year to < 5 years (25.4%), with slightly more in the groups 5 years to < 10 years (34.2%) and 10 years to < 20 years (28.9%). Participants with ≥ 20 years of experience in the same facility comprised 11.4%. Although there was no significant difference in the length of employment between participants from MOUs and DHs (*p* = 0.1778), there were proportional differences amongst specific employment groups. For example, DHs had more participants in the 1 year to < 5 years age group (30.6%) than MOUs (21.5%) and in the ≥ 20 years age group (18.4% vs. 6.2%). On the other hand, MOUs had more than double the number of participants in the 10 years to < 20 years age group (38.5%) than DHs (16.3%).

### Perceived changes in well-being

For all four items that measured perceived changes in well-being, there was a significant change in the median score out of 10 that participants gave themselves for the ‘before’ and ‘during’ COVID-19 periods (*p* < 0.0001 for all four). Table 1 provides an overview of the ‘before-during’ COVID-19 differences in perceived feelings of well-being. The biggest ‘before-during’ difference was in perceptions of fear or anxiety and the smallest difference was in perceptions of anger. Anger also scored the lowest in the ‘before’ and ‘during’ COVID-19 periods, with stress scoring the highest in both periods.

**TABLE 1:** Self-reported change in overall perceptions of well-being before and during the COVID-19 pandemic.

Well-being term	Median score (out of 10)			*p*
	Before COVID-19	During COVID-19	Difference	
Fear/Anxiety	2.00 (0.00, 4.00)	9.00 (7.00, 10.00)	6.00 (4.00, 8.00)	< 0.0001
Stress	3.00 (1.00, 5.00)	9.00 (8.00, 10.00)	5.00 (3.75, 7.00)	< 0.0001
Depression	1.00 (0.00, 4.00)	8.00 (4.00, 10.00)	4.00 (1.00, 7.00)	< 0.0001
Anger	1.00 (0.00, 3.00)	7.00 (3.00, 10.00)	4.00 (0.25, 7.00)	< 0.0001

**Overall**	**1.75 (0.75, 3.88)**	**7.75 (5.75, 9.00)**	**5.00 (3.00, 7.00)**	**< 0.0001**

**FIGURE 1:**
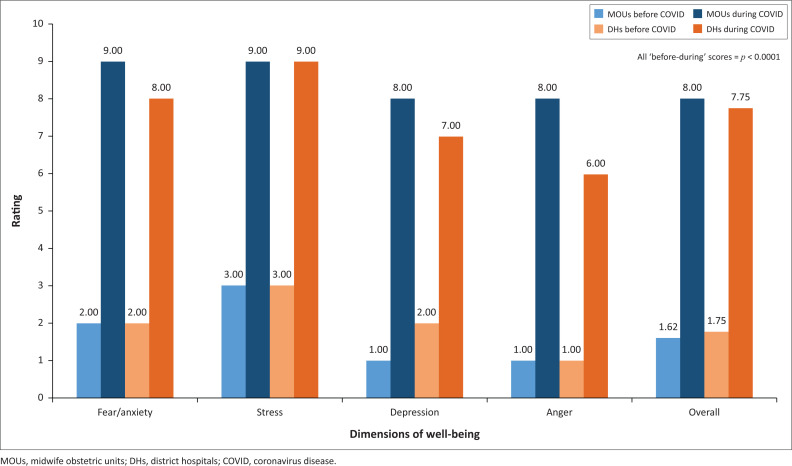
Changes in perceptions of well-being of participants in midwife obstetric units and district hospitals.

There were no significant differences between MOU and DH participants’ perceptions of well-being for fear or anxiety, stress, depression and anger before the COVID-19 pandemic nor between their perceptions after the advent of COVID-19 (*p* = 0.3364). Figure 1 depicts the median ‘before’ and ‘during’ scores. There was a tendency for MOU participants to rate themselves higher on their perceptions of depression than DH participants during the COVID-19 period (*p* = 0.0730). Participants from MOUs had higher negative perceptions during the COVID-19 period than their counterparts in DHs with regard to anger, but this difference was not significant (*p* = 0.1030).

**FIGURE 2:**
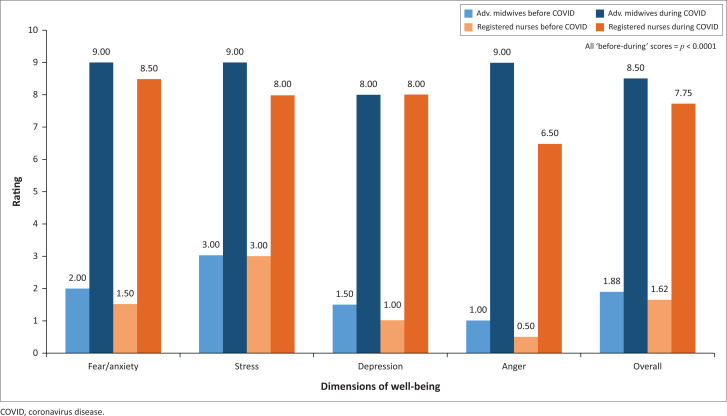
Changes in perceptions of well-being in advanced midwives and registered nurses.

There was no significant difference between participants of different designations with regard to their change in perceptions of well-being before and after the advent of COVID-19 (*p* = 0.1174), although there was a trend towards a greater negative change in advanced midwives’ and registered nurses’ perceptions of depression (*p =* 0.0723) and anger (*p* = 0.0529). Because of the small number of respondents in some designation categories, we have only provided a visual representation of the changes in median perception scores for advanced midwives and registered nurses (see Figure 2).

Age of participants did not play a significant role in the change in perceptions of well-being from before to during the COVID-19 pandemic (*p* = 0.4792). There was also no significant difference between length of employment at a particular health facility and change in perceptions of well-being over time (*p* = 0.1441).

### Reasons for changes in perceived well-being

In the open-ended item in which participants were requested to give their reasons for their ratings on their perceived well-being, some participants responded by directly referring to their well-being in terms of fear or anxiety, stress, depression and anger. Others used more general descriptions as a justification.

**FIGURE 3:**
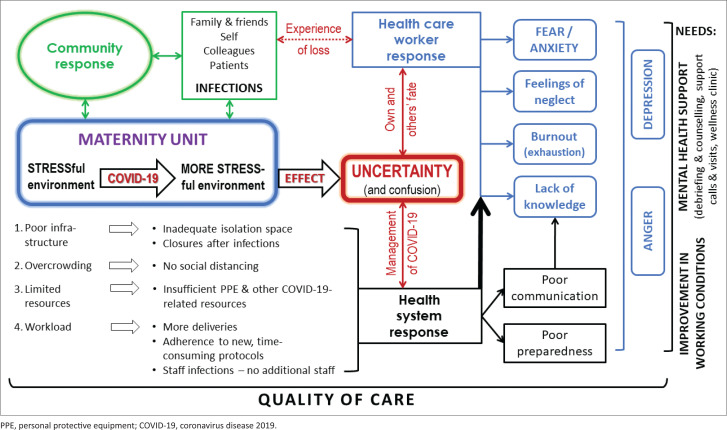
Reconstruction of healthcare workers’ understandings and perceptions of the causes of their increased negative feelings regarding mental well-being.

We constructed a framework to explain negative change in HCWs’ perceptions of their own mental well-being, which is depicted in Figure 3. The main themes depicted in this figure relate to the stressful environment of the maternity unit, the uncertainty emanating from the stressful environment (red block in the middle of the figure) and then the responses of the community (green), the health system (black) and the HCWs (blue) to the pandemic. All of these issues affect the quality of care (depicted at the bottom of the figure). On the right-hand side of the figure, the needs expressed by participants are observed (What could have been done differently?). In direct quotations below, references to MOUs are given as ‘M’ plus ‘A’ to ‘J’ for each particular facility and ‘H’ followed by ‘A’ to ‘D’ for each DH. The number that follows refers to the respondent number for that particular facility. ‘AM’ refers to an advanced midwife as respondent, ‘RN’ to a registered nurse-midwife, ‘ENA’ to an enrolled nursing assistant and ‘M’ to a manager.

#### Working in a stressful environment

Maternity units are by nature a stressful working environment. Participants referred to the pre-COVID-19 era regarding infrastructure, overcrowding, limited resources and a heavy workload, but they also described the deterioration of working conditions with the advent of COVID-19:

‘Before COVID-19 our situation in facility was uncomfortable and with COVID-19 situation becomes worse.’ (MD07, AM)

Reasons for the increase in stress levels included challenges in protecting staff and patients, patients not adhering to COVID-19 rules, communication difficulties with patients, lack of space for accommodating COVID-19 positive patients and an increase in health workers’ workload:

‘During COVID-19 the situation was so hectic and even now because the patients don’t want to put on the mask during delivery, they are stressing us, no support person [*labour companion*] allowed, some patients communication breaks down. COVID-19 really its stressing midwives!!!’ (ME04, AM)

Inadequacies in *infrastructure* became glaringly obvious after the start of the pandemic:

‘Having to work under stress, being understaffed in a limited area [*infrastructure*], no designated area for pregnant PUI’s. We have admitted positive patients in the same ward where non-suspects were kept so it was really stressful for both staff and non-suspect [*patients*].’ (HA07, M)

Infrastructure is related to inadequate space and the accompanying *overcrowding* of facilities. Overcrowding made it impossible to practise social distancing – ‘Since COVID pandemic we are unable to social distance and the ward is very, very busy’ (MH08, AM). Delivering a baby is by nature not conducive to maintaining social distancing ‘I’m afraid of COVID-19 because … [*it*] is not possible for us during delivery of a patient for social distancing’ (HC14, RN).

Inadequacy of *resources* for health workers to maintain occupational safety was the topic participants mentioned most often. The main issue was the use and availability of PPE – ‘I was not provided with PPE during the pandemic’ (HB07, AM). Unavailability of sufficient numbers of masks was highlighted – ‘We wear one mask for the whole day, which puts us at risk of COVID-19’ (MA12, RN). The wearing of masks was also a source of frustration: ‘I feel overworked and frustrated by the fact that I have to wear a mask and I feel like I am suffocating’ (MC02, RN). In addition, some patients contributed to the situation because they ‘did not even use masks, presenting in advanced labour’ (HC01, M).

Increase in *workload*, the ‘sudden increase in the amount of work’ (HD13, M), was another prominent reason for participants’ change in perceptions of their own mental well-being: ‘COVID-19 requires extra human and other resources that we don’t have in the facility and this causes the present human resource to be overwhelmed with the current situation’ (MA10, RN). There were a number of causes for this situation. The new admission and treatment protocols for all patients, for PUIs and for COVID-19 confirmed patients slowed down care because the actions required extra time for compliance. Despite the additional screening measures, ‘sometimes it’s hard to screen clients who are already in labour’ (MD04, AM). Participants felt unsafe because ‘I concentrate on saving lives of babies and do screening later’ (HC12, RN).

Additional overcrowding was caused by ‘increased number of deliveries’ (MH01, AM) and referral of patients from other facilities for antenatal care or delivery, for example, ‘influx of patients when other clinics are fumigated’ (MH05, AM) and ‘more work has been expected of us without extra staff’ (MA11, AM). Moreover, staff shortages were aggravated when staff members contracted the coronavirus or ‘were under quarantine’ (HA03, AM).

#### Effect of the stressful working environment: Uncertainty

Uncertainty ‘of the future’ (HD13, M) is a key lens for understanding HCWs’ perceptions of the change in their own mental well-being. Uncertainty is related to the response of the health system on the one hand and HCWs’ response to the pandemic on the other.

The health system needed to adapt to the management of the pandemic with ‘frequent changes in protocol and management’ (HC08, AM), which left the HCWs with feelings of helplessness:

‘The stress level had increased since COVID-19 started because we don’t even know how to manage COVID-19 patients.’ (ME03, RN)

‘I felt so overwhelmed, lost and fearful. Everything changed and I was expected to adapt quickly and mostly I felt it was with trial and error as COVID was a new thing.’ (HB08, AM)

The HCWs’ response pivoted around their own ‘fate’ with regard to contracting the virus and the ‘fate’ of others:

‘Constantly living in fear of being infected with COVID-19 and not knowing what the outcome may be.’ (MF06, AM)

#### The community’s response to the pandemic

All interactions reported in this study between a health facility and its HCWs, on the one hand, and patients and the community at large, on the other, revolved around coronavirus infections and the behaviour of community members. ‘Ignorance of the community towards regulations’ (MD05, AM) and ‘patients not understanding the rules of COVID’ (MA07, M) were an issue for some participants. Stigmatisation of HCWs was also mentioned:

‘Outside workplace, community is afraid of a HCWs they think we are carriers of COVID-19.’ (HD16, AM)

At the interface between the community’s response and its interaction with the maternity unit was HCWs’ *experience of loss* of colleagues (health system) and family and friends (community) as a result of coronavirus infections – ‘I don’t know what tomorrow brings because a lot of people are getting infected by coronavirus more especially after losing my sister and colleague recently’ (MF02, AM). The effect of loss was also described as follow:

‘Losing a colleague from COVID-19 when you were working with her caused so much stress. I was scared I will be next. I got infected so it was scary hearing a colleague died.’ (MF04, RN)

#### The health system’s response to the pandemic

Participants’ perceptions of the health system’s perceived failed response to the pandemic was linked to the deteriorating working conditions in individual facilities and to ‘lack of information and preparedness about COVID-19’ (HC08, AM) – ‘The whole situation and how it was handled in the facility was just not helping us as health workers’ (HC14, RN).

The health system’s poor communication with frontline workers – ‘no information and reassurance’ (HC14, RN) – resulted in lack of knowledge: ‘We were not taught or workshopped on the COVID-19. No guidelines were given in time’ (MJ04, RN). ‘I had a stress with regard to our PPE and how we manage patient with COVID-19 in our unit’ (HC02, ENA). Participants also ‘felt unsupported … when colleagues tested positive in the unit and not disclosing the tested positive staff’ (MI05, RN).

The various response failures of the system fed into the change in the mental well-being of participants: ‘I believe uncertainty and a lack of communication from management during the times that the hospital closed down because of COVID-19 played a big role in my increased anxiety and depression’ (HA10, RN).

#### Healthcare workers’ response to the pandemic

Uncertainty, the poor health-system response, the stigmatising community response to the pandemic and experience of loss were all reported as having a bearing on the deteriorating mental well-being of frontline workers. Three main negative feelings dominated HCWs’ response to the pandemic: fear and anxiety, feelings of neglect and feelings of burnout. These in turn fuelled perceptions of depression and anger.

Participants described their *fear and anxiety* in the form of a chain of events: contact with patients may lead to own infection, which in turn may lead to infection of family and friends – ‘Fears of infection transmission to my kids and close family members because of contact with patients’ (HC04, RN). All thoughts were permeated with uncertainty. Participants were ‘scared of what is going to happen to me’ (ME05, AM) with regard to their own health or death (e.g. ‘because of comorbidity’ [MH05, AM]). This is how one participant described her state of mind: ‘Feelings of fearing contracting COVID-19 dying and leave my family members who will suffer in my absence’ (ME06, AM).

Participants expressed their feelings of being *neglected* and *burnt out* as ‘exhausted BUT not appreciated’ (HD12, AM). Perceived neglect and non-appreciation by the employer were at the forefront. Neglect focused on the poor working conditions such as the absence of proper provision of PPE and other resources – ‘we put our lives at risk’ (HC14, RN) – and the work overload. Furthermore, ‘lack of support from the employer’ (HB13, RN) and ‘no emotional support and appreciation’ (HB01, RN) led to the perception of participants’ ‘psychological, emotional and physical need [*being*] abandoned’ (MD02, AM).

*Anger* and *depression* were caused by ‘working condition’ (MD04, AM) that put participants and their ‘families at risk, with no compensation or remuneration whatsoever’ (HD15, RN). This was aggravated by experiences of loss – ‘depressed and angry because I have lost loved ones because of COVID-19 in family, colleagues’ (MF06, AM) – and ‘fear of dying increased my anxiety to the extent that I’m now on anti-depression drugs’ (MI03, AM).

#### Quality of care at stake

In the complex context of the maternity unit, many interrelated actions and behaviours influenced the quality of care – ‘COVID-19 has impacted our standard of care as it took all our resources and focused on COVID-19’ (MD06, RN). One participant also referred to a ‘lack of support system from the clinical management’ (MH01, AM) and another noted:

‘First happened in health facility, no one knows what to do and it seems victims were very anxious and some mismanagement was done to victims. Fear of death was amongst victims.’ (HA01, AM)

Patients were no longer allowed to have visitors or birth companions to support women during childbirth, ‘which leads to young primigravida not responding when questions are asked’ (HB04, AM). In addition, social distancing and health workers’ fear of contracting the virus meant ‘less contact with patients’ relatives and patient’ (HB03, RN). ‘Instead of looking at patients with a caring eye, I look at them as someone who can make me sick’ (MD01, RN).

#### What could have been done differently?

Study participants had two prominent wishes regarding what they would have liked to be different. The one is an improvement in working conditions and the other is appropriate mental health support.

Participants’ need for *improved working conditions* included having ‘a proper plan and the plan must be consistent’ (HC14, RN), providing ‘more PPE’ (MH04, RN) and ‘a proper clinic … without structural constraints’ (MG06, AM). Good communication and proper information also featured strongly: ‘how to deal with the pandemic’ (HC06, RN) and ‘correct procedure to follow during the pandemic’ (MB02, RN). Training was mentioned with regard to ‘management of COVID-19 patients’ (ME03, RN), ‘continuous support in patient care strategies’ (HC11, M), ‘PPE training for all staff’ (MA10, RN), and ‘being taught every day about the COVID-19 to be able to know it in detail’ (HB08, AM). Some participants felt that they should get ‘compensation for all the dedication and hard work’ (HC07, RN).

With regard to *mental health support,* participants used a variety of expressions to describe their needs. There was a desire for ‘acknowledgement of HCWs’ (MC04, RN) and ‘appreciation for all the best we are trying to give to the community’ (HB01, AM). Support was described as ‘moral support’ (HB09, ENA) and ‘psychological support’ (MF08, AM). A manager referred to emotional support:

‘I think emotional support is always important for health workers. They are also social beings who over and above also experience challenges in their personal life – in addition to the work-related challenges.’ (HB14, M)

The need for debriefing, including individual counselling, was the most prominent action desired from employers – for general support, after a HCW had tested positive for COVID-19 or after the loss of a colleague or family member. Participants noted:

‘Debriefing of health staff, continuous understanding and attending to the way health professionals feel and the trauma that [*they experience*] towards this pandemic.’ (MD01, RN)

‘Psychologist must come and do debriefing – even after two staff members died, the district only did group counselling, which is not enough.’ (MF04, RN)

‘Support visit for helping us, not inspecting’ (MA10, RN) and a ‘telephone call to ask how are you coping with the pandemic would have been much appreciated’ (HD17, M). Especially staff who had tested positive needed ‘checking up through calls and messages to reassure them’ (MI02, AM).

## Discussion

The ongoing COVID-19 pandemic with surges and isolation has placed enormous pressure on HCWs around the world, and thereby has affected their mental well-being. Participants in our study reported significant changes in their perceived well-being from the pre-pandemic to the pandemic phase with regard to fear or anxiety, stress, depression and anger. Worldwide, working on the frontline was associated with a higher risk of mental health disturbance amongst HCWs.^5,9,12,17^ Chinese researchers, especially from Wuhan where the pandemic started, reported a high prevalence of symptoms of depression, anxiety, insomnia and distress, which they considered to be the result of social isolation and self-perceived stress.^12,14,15,29^

The reasons other researchers cited for the deterioration in mental health during the pandemic are similar to those found in our study. Study participants described how their stressful environment became more stressful over time and how essential services deteriorated because of poor infrastructure, overcrowding, limited resources such as PPE and increased workload. They expressed a need for improved working conditions and support to keep the health system functioning with regard to the maintenance of the supply chain and essential services, echoing similar voices from LMICs.^3,19^ In other studies, environment stressors like increased patient loads and high risk of exposure together with insufficient PPE and lack of resources were associated with a 46% or higher increase in the risk of combined depression and anxiety.^17,30^ In Tshwane Health District, increased deliveries resulted in overcrowding because of the change of some the delivery sites to COVID-19 facilities and temporary closures of others for deep cleaning after staff had contracted the virus. Our HCWs also explained how the new admission protocols and guides for PUIs slowed down care and said that despite additional screening measures, they feared for their own safety and did not always feel protected. Similarly, new guidelines and policies in other countries were not discussed with staff, whilst poor communication with little input from these frontline HCWs on implementing these changes led to mistrust and an altered sense of security amongst nurses.^16,31^

Participants’ biggest need was appropriate mental health support. Their increased negative feelings regarding aspects of their mental well-being were highlighted in the direct quotations from their statements, which brought their lack of knowledge, fear and anger, feelings of neglect, exhaustion and burnout to the forefront. Elsewhere, and in our study, healthcare providers’ inability to maintain family and community life as a result of the fact that they could transmit infections^32,33^ and the perceived stigma on the part of family and society compounded their distress.^12,16,30^ Participants’ responses demonstrated suboptimal resilience and uncertainty about the future during the surge of the pandemic, which is also mirrored in other countries.^4,10,15^

In many countries and in Tshwane District, the community and health workers’ response to the pandemic influenced the quality of care and the constant fear of contracting the virus impacted on their functioning in the workplace.^8^ They pleaded for clear communication with added training and clear protocols, information sharing and outreach support to healthcare facilities from the institution and management, as well as for reassurance and debriefing.^7^

Healthcare workers in our study expressed the need for moral and psychological support and acknowledgement of hard work and dedication. They proposed a demonstration of interest by health managers in their well-being, inter alia through debriefing opportunities to communicate and work through their feelings and traumatic experiences. Pandemic preparedness entails improved clinical governance and accountability that include effective psychosocial support in teams.^15,34^ Psychosocial support can be divided into the following: self-coping strategies (e.g. exercise and self-help resources through the media), interventions that promote well-being (e.g. team collaborations, training and education), and interventions to address stigma (e.g. clearer disease information and how to handle isolation).^12,14,29^ Communities need to support their nurses, whilst managers must attend to modifiable elements that would relieve the mental health burden of all frontline health workers and nurses.^9,11,16,34^ Strategies that team leaders and managers could consider for relieving depression and anxiety in clinical staff include: reducing the chances of infection, shorter shift lengths and mental health support via outreach, phoning in, sharing information and debriefing (e.g. conversational sessions to address HCWs’ psychological self-care).^5,14,29,34^

Resilient strengthening teams, encouraging sharing of distress and enhancing self-compassion can assist individuals in need.^34^ People need time to process their concerns and feelings, especially after their own infection with COVID-19 or the loss of a colleague. To achieve this, social connection, the use of buddy systems and individual psychological support services should be encouraged in all facilities.^34^

## Study limitations

This study entailed a small investigation with a convenience sample of maternity HCWs in one district in South Africa. It was meant as a rapid assessment to inform Tshwane District Management on the state of affairs in the district and on what could be done to support frontline maternity workers. As a result of the urgency of the pandemic, it was not possible to pilot the application of the tool. The results of the analysis of the open-ended question may not be generalisable to other districts. However, our findings are very similar to the findings of other studies conducted in different contexts and may be transferable to similar settings elsewhere.

## Conclusion

This article provides a small snapshot at the primary care level in one health district in South Africa of healthcare providers’ self-perceptions of their mental well-being before and during the COVID-19 pandemic. These findings could be useful in planning, especially with regard to improved health-systems functioning, appropriate mental health support with debriefing and ongoing communication with information sharing. In the case of resurgence of infections, the same type of methodology could be applied at different times in this pandemic to observe trends in the change of HCWs’ self-perceptions of their mental well-being and to act immediately where necessary.

## References

[1] Abdelbadee AY, Abbas AM. Impact of COVID-19 on reproductive health and maternity services in low resource countries. Eur J Contracept Reprod Health Care. 2020;25(5):402–404. 10.1080/13625187.2020.1768527 32436744

[2] Frawley T, Van Gelderen F, Somanadhan S, et al. The impact of COVID-19 on health systems, mental health and the potential for nursing. Ir J Psychol Med. 2020;38(5);220–226. 10.1017/ipm.2020.105 32933594 PMC7596574

[3] Semaan A, Audet C, Huysmans E, et al. Voices from the frontline: Findings from a thematic analysis of a rapid online global survey of maternal and newborn health professionals facing the COVID-19 pandemic. BMJ Glob Health. 2020;5(6):e002967. 10.1136/bmjgh-2020-002967 PMC7335688 32586891

[4] Mira JJ, Carrillo I, Guilabert M, et al. Acute stress of the healthcare workforce during the COVID-19 pandemic evolution: A cross-sectional study in Spain. BMJ Open. 2020;10(11):e042555. 10.1136/bmjopen-2020-042555 PMC7650075 33158839

[5] Shaukat N, Ali DM, Razzak J. Physical and mental health impacts of COVID-19 on healthcare workers: A scoping review. Int J Emerg Med. 2020;13:40. 10.1186/s12245-020-00299-5 32689925 PMC7370263

[6] Garg S, Basu S, Rustagi R, Borle A. Primary health care facility preparedness for outpatient service provision during the COVID-19 pandemic in India: Cross-sectional study. JMIR Public Health Surveill. 2020;6(2):e19927. 10.2196/19927 32452819 PMC7265797

[7] Afulani PA, Gyamerah AO, Aborigo RA, et al. Perceived preparedness to respond to the COVID-19 pandemic: A study with healthcare workers in Ghana. J Glob Health Sci. 2020;2(2):e24. 10.35500/jghs.2020.2.e24

[8] Pollock A, Campbell P, Cheyne J, et al. Interventions to support the resilience and mental health of frontline health and social care professionals during and after a disease outbreak, epidemic or pandemic: A mixed methods systematic review. Cochrane Database Syst Rev. 2020;Issue 11:Art. No: CD013779. 10.1002/14651858.CD013779 PMC8226433 33150970

[9] AlAteeg DA, Aljhani S, Althiyabi I, Majzoub S. Mental health among healthcare providers during coronavirus diseade (COVID-19) outbreak in Saudi Arabia. J Infect Pub Health. 2020;13(10):1432–1437. 10.1016/j.jiph.2020.08.013 32933881 PMC7834809

[10] Carmassi C, Foghi C, Dell’Oste V, et al. PTSD symptoms in healthcare workers facing the three coronavirus outbreaks: What we can expect after the COVID-19 pandemic. Psychiatry Res. 2020;292:113312 10.1016/j.psychres.2020.113312 32717711 PMC7370915

[11] Azoulay E, Cariou A, Bruneel F, et al. Symptoms of anxiety, depression and peritraumatic dissociation in critical care clinicians managing COVID-19 patients: A cross-sectional study. Am J Respir Crit Care Med. 2020;2021(10):1388–1398. 10.1164/rccm.202006-2568OC PMC7667906 32866409

[12] Cabarkapa S, Nadjidai SE, Murgier J, Ng CH. The psychological impact of COVID-19 and other viral epidemics on frontline healthcare workers and ways to address it: A rapid systematic review. Brain, Behav Immun – Health. 2020;8:100144. 10.1016/j.bbih.2020.100144 32959031 PMC7494453

[13] Robertson LJ, Maposa I, Somaroo H, Johnson O. Mental health of healthcare workers during the COVID-19 outbreak: A rapid scoping review to inform provincial guidelines in South Africa. S Afr Med J. 2020;110(10):1010–1019. 10.7196/SAMJ.2020.v110i10.15022 33205731

[14] Ruiz-Fernández MD, Ramos-Pichardo JD, Ibáñez-Masero O, Cabrera-Troya J, Carmona-Rega MI, Ortega-Galán MA. Compassion fatigue, burnout, compassion satisfaction and perceived stress in healthcare professionals during the COVID-19 health crisis in Spain. J Clin Nurs. 2020;29(21–22):4321–4330. 10.1111/jocn.15469 32860287

[15] Chen H, Sun L, Du Z, Zhao L, Wang L. A cross-sectional study of mental health status and self-psychological adjustment in nurses who supported Wuhan for fighting against the COVID-19. J Clin Nurs. 2020;29:4161–4170. 10.1111/jocn.15444 32757428 PMC7436217

[16] Sharma M, Creutzfeldt CJ, Lewis A, et al. Healthcare professionals’ perceptions of critical care resource availability and factors associated with mental well-being during COVID-19: Results from a US survey. Clin Infect Dis. 2020;72(10):e566–e576. 10.1093/cid/ciaa1311 PMC7499503 32877508

[17] Suryavanshi N, Kadam A, Dhumal G, et al. Mental health and quality of life among healthcare professionals during the COVID-19 pandemic in India. Brain Behav. 2020;10(10):e01837. 10.1002/brb3.1837 32918403 PMC7667343

[18] Blanchet K, Alwan A, Antoine C, et al. Protecting health services in low-income and middle-income countries and humanitarian settings while responding to the COVID-19 pandemic. BMJ Glob Health. 2020;5:e003675. 10.1136/bmjgh-2020-003675 PMC7542611 33028701

[19] Nyasulu J, Pandya H. The effects of coronavirus disease 2019 pandemic on the South African health system: A call to maintain essential health services. Afr J Prim Health Care Fam Med. 2020;12:e1–e5. 10.4102/phcfm.v12i1.2480 PMC7433230 32787396

[20] Bhaumik S, Moola S, Tyagi J, Nambiar D, Kakoti M. Community health workers for pandemic response: A rapid evidence synthesis. BMJ Glob Health. 2020;5:e002769. 10.1136/bmjgh-2020-002769 PMC7292038 32522738

[21] Chen T, Wang Y, Hua L. ‘Pairing assistance’: The effective way to solve the breakdown of health services system caused by COVID-19 pandemic. Int J Equity Health. 2020;19:68. 10.1186/s12939-020-01190-8 32414384 PMC7226711

[22] Sarwer A, Javed B, Soto EB, Mshwani Z. Impact of the COVID-19 pandemic on maternal health services in Pakistan. Int J Health Plann Manage. 2020;35(6):1306–1310. 10.1002/hpm.3048 32869363

[23] Petterson J, Jonson C-O, Berggen P, et al. Connecting resillience concepts to operational behaviour: A disaster exercise study. J Contingencies Crisis Manag. 2021. 10.1111/1468-5973.12373

[24] Wiig S, Aase K, Billet S, et al. Defining the boudaries and operational concepts of resilience in the resilience in healthcare research program. BMC Health Serv Res. 2020;20(1):330. 10.1186/s12913-020-05224-3 32306981 PMC7168985

[25] Khetrapal Singh P, Jhalani M. Safeguarding essential health services during emergencies: Lessons learnt from the COVID-19 pandemic. WHO South-East Asia J Public Health. 2020;9(2):93–94. 10.4103/2224-3151.293433 32978338

[26] Kringos D, Carinci F, Barbazza E, et al. Managing COVID-19 within and across health systems: Why we need performance intelligence to coordinate a global response. Health Res Policy Syst. 2020;18:80. 10.1186/s12961-020-00593-x 32664985 PMC7358993

[27] Oosthuizen SJ, Bergh A-M, Grimbeek J, et al. Midwife-led obstetric units working ‘CLEVER’: Improving perinatal outcome indicators in a South African health district. S Afr Med J. 2019;109(2):95–101. 10.7196/SAMJ.2019.v109i2.13429 30834859

[28] R Core Team. R: A language and environment for statistical computing [homepage on the Internet]. Vienna: R Foundation for Statistical Computing. Available from: https://www.R-project.org/

[29] Lai J, Ma S, Wang Y, et al. Factors associated with mental health outcomes among health care workers exposed to coronavirus disease 2019. JAMA Netw Open. 2020;3(3):e203976. 10.1001/jamanetworkopen.2020.3976 32202646 PMC7090843

[30] Şahin MK, Aker S, Şahin G. Prevalence of depression, anxiety, distress and insomnia and related factors in healthcare workers during COVID-19 pandemic in Turkey. J Community Health. 2020;45:1168–1177. 10.1007/s10900-020-00921-w 32915381 PMC7485427

[31] Owens IT. Supporting nurses’ mental health during the pandemic. Nursing. 2020;50(10):54–57. 10.1097/01.NURSE.0000697156.46992.b2 32947374

[32] Yang S, Kwak SG, Chang MC. Psychological impact of COVID-19 on hospital workers PTSD symptoms in healthcare workers acing the three coronavirus outbreaks: What can we expect after the COVID-19 pandemic. Psychiatry Res. 2020; 292:113312. 10.1016/j.psychres.2020.113312 32717711 PMC7370915

[33] Rose S, Hartnett J, Pillai S. Healthcare worker’s emotions, perceived stressors and coping mechanisms during COVID-19 pandemic. PLos One. 2021;16(70):e0254252. 10.1371/jounal.pone.025452 34242361 PMC8270181

[34] Tomlin J, Dalgleish-Warburton B, Lamph G. Psychosocial support for healthcare workers during the COVID-19 pandemic. Front Psychol. 2020;11:1960. 10.3389/fpsyg.2020.01960 32849149 PMC7431467

